# Does the establishment of civilized cities promote urban inclusive development?——Quasi-natural experiment based on the selection of “National Civilized City”

**DOI:** 10.1371/journal.pone.0307645

**Published:** 2024-12-12

**Authors:** Enguang Zhang, Jing Zhang, Xuesi Li, Yangyang Dai, Mingdou Zhang, Weilu Li

**Affiliations:** 1 School of Economics and management, Chongqing Three Gorges Vocational College, Chongqing, China; 2 School of Economics, Dongbei University of Finance and Economics, Dalian, China; 3 Institute for Northeast Full Revitalization, Dongbei University of Finance and Economics, Dalian, China; 4 Center for Industrial and Business Organization, Dongbei University of Finance and Economics, Dalian, China; Wuhan Institute of Technology, CHINA

## Abstract

Taking the selection of “National Civilized City” as a quasi-natural experiment and basing on the research samples of 272 prefecture level and above cities in China from 2003 to 2020, this paper explores the impact of the establishment of civilized cities on urban inclusive development and its mechanism by using the time-varying *DID* model. The findings are as follows: firstly, the establishment of civilized cities has significantly promoted the urban inclusive development, and then parallel trend test, placebo test and other robustness tests verify the reliability of the research conclusions. Secondly, in regions with larger population, stronger financial strength and belonging to the Excellent Tourism City, the establishment of civilized cities has a more obvious role in promoting urban inclusiveness. Thirdly, the establishment of civilized cities promotes the urban inclusive development through government public regulation at the macro level, industrial structure upgrading at the middle level, and talent gathering and infrastructure construction at the micro level. The research conclusion of this paper has a certain reference value for giving full play to the policy effect of the selection of “National Civilized Cities”, further promoting the urban inclusive development and achieving common prosperity.

## 1. Introduction

Over the past 40 years of reform and opening up, China’s urbanization construction had been rapidly advancing and economic and social development had created a world-renowned “China miracle”. However, due to historical reasons and institutional mechanisms, many problems and crises had been exposed behind the rapid advancement of urbanization, such as weak economic growth, unequal development opportunities, and difficulty in sharing development results [1]. At the same time, the extensive and aggressive development model had caused a series of practical problems such as excessive consumption of resources, deterioration of the ecological environment, and widening wealth gap, seriously constraining the healthy process of urbanization. In the context of China’s overall promotion of common prosperity, the proposal and implementation of the concept of “inclusive development” provided feasible solutions to the above prominent contradictions and problems [2, 3]. The Outline of the 13-th Five Year Plan for National Economic and Social Development of the People’s Republic of China pointed out that “The government should promote the inclusiveness of development through economic co construction and sharing”. It can be seen that how to implement the concept of “inclusive development” and achieve urban inclusive development had become an important goal and fundamental task of China’s economic and social development, and was also an important topic worth studying at present.

Civilization played a commanding and leading role in the entire socio-economic system, and consolidating the quality of urban development and improving the level of urban civilization had become an inevitable choice for urban development. As the highest honor title reflecting China’s urban civilization construction, the “National Civilized City” had undergone a total of 6 rounds of selection since its launch in 2005, covering 146 prefecture level and above cities in 31 provinces (cities, districts) across the country. The implementation of this selection activity has greatly optimized the overall layout of the “Five in One”, improved the level of civilization of the city, improved the development environment of the city, and provided guarantees for the harmonious development of people, nature, and society. The gradually improved evaluation system in the process of selecting civilized cities had put forward many requirements for the development and humanistic literacy of participating cities, which to some extent will also enhance the equality of development opportunities, comprehensiveness of development content, and sharing of development achievements of cities, which helped to promote the achievement of inclusive urban development. However, has the construction of civilized cities effectively promoted inclusive urban development? What is the mechanism of its impact? There is still a lack of systematic research to explain such issues. Therefore, based on the above analysis, this paper attempts to investigate the policy effect of the selection of “National Civilized Cities”, and identify the internal mechanism between the construction of civilized cities and urban inclusive development, which is of great practical significance for consolidating the construction achievements of “National Civilized Cities”, improving the quality of urbanization development, and achieving common prosperity.

## 2. Literature review

The literature closely related to this study mainly involved the policy effects of the “National Civilized City” selection and the influencing factors of urban inclusive development.

### 2.1 The policy effect of the “National Civilized City” selection

Currently, the proposal of the “National Civilized City” selection had attracted widespread attention from scholars. From the perspective of enterprises, Wu et al. [4] were the first to use the “National Civilized City” selection policy to construct a quasi-natural experiment, based on the kernel matching multiple difference method to examine the impact of urban civilization on corporate profit margins, and they found that urban civilization increased the profit level of enterprises by reducing market transaction costs. However, Zheng and Zhang [5] concluded a contradictory conclusion, and they pointed out that the construction of civilized cities places higher demands on the ecological environment and technological innovation of enterprises, and the increase in production costs inhibits the improvement of enterprise profit margins. In addition, other scholars had pointed out that the construction of civilized cities had improved corporate environmental performance [6], enhanced corporate social responsibility [7], and strengthened corporate emission reduction through local government incentive measures [8]. From a regional perspective, the construction of civilized cities can help improve the level of urban green innovation [9, 10], enhance green total factor productivity and green total factor carbon efficiency [11–13], promote the sustainable development of Chinese cities [14], and the development of local domestic and international tourism industry [15], continuously attract foreign direct investment [16]. Meanwhile, in terms of environmental effects, the construction of civilized cities had significantly reduced the concentrations of PM2.5, SO_2_, and CO_2_ [17], reduced carbon emissions [18], and optimized the ecological environment of the city. From the perspective of the honorary title of “European Civilization Capital”, Gomes and Librero Cano [19] found that winning the title of “European Civilization Capital” increased the per capita GDP of the region and promoted urban renewal and development; However, Steiner et al. studied the impact of the “European Capital of Civilization” on economic growth and residents’ satisfaction, and pointed out that the title did not exert an economic growth effect, but instead reduced the welfare level of local residents [20]; Falk and Hagsten [21] pointed out that the title only significantly increased the number of overnight tourists in the short term and did not have a long-term effect.

### 2.2 The influencing factors of urban inclusive development

Since the Asian Development Bank first proposed “inclusive growth”, domestic and foreign scholars have conducted corresponding research on its influencing factors from the perspective of empirical deduction, mainly focusing on the following three levels. From a macro perspective, Anand et al. [22] first measured the level of inclusive development in various countries and pointed out that economic stability and sufficient human capital were the foundations for achieving inclusive growth. Foreign direct investment and trade openness can promote inclusive growth. Whajah et al. [23] pointed out that government size had a positive impact on inclusive growth, but the level of public debt has a negative impact on inclusive growth; In addition, the improvement of regional coordination mechanisms, the increase in public education expenditures, and the expansion of urban land all contribute to narrowing the urban-rural income gap and promoting inclusive urban development [24–26]. From a meso perspective, Meng et al. [27] focused on the circulation industry and found that the informationization development of the circulation industry can coordinate urban and rural, social and economic development, and thereby enhance the level of inclusive development. Chen and Qin [28] pointed out that under the effects of job turnover and productivity, artificial intelligence had generally promoted inclusive growth within the industry, narrowing the labor income gap among different classes, based on world industrial robot installation data at the industry level. At the micro level, He and Du [29] and Corrado and Corrado [30] pointed out that digital inclusive finance can promote inclusive development, but urbanization and inclusive finance weaken each other when narrowing income disparities. Ofori and Asongu [31] found that foreign direct investment contributes to inclusive development in regions, and by combating corruption and improving government structures, this effect can be promoted. Jia et al. [32] pointed out that innovation has become a key factor in balancing economic growth and social inclusion. Among them, independent innovation had a positive impact on inclusive growth, while imitation innovation has a negative impact.

In a word, although existing research has systematically and deeply explored the policy effects of the selection of “National Civilized Cities” from the perspective of enterprises, regions and governments, under the situation of the country’s solid promotion of common prosperity, there was a lack of empirical analysis on urban inclusive development, and research on evaluating the impact of a policy on urban inclusive development was even more minimal. In fact, civilization, as the core competitiveness of urban development, subtly influences the inclusive development of cities through human civilization, material civilization, and institutional civilization. Thus, a systematic evaluation of the impact of civilized city construction on inclusive development will be sufficient as the starting point and foothold of this study.

The possible marginal contributions of this article mainly lie in: From a research perspective, this article examined the relationship between the construction of civilized cities and urban inclusive development by constructing a quasi-natural experiment for the selection of “National Civilized Cities”, which expanded the research on the policy effects of the selection of “National Civilized Cities” and further enriched the theoretical system of inclusive development. In addition, the investigation verified the effectiveness of promoting inclusive urban development through the analysis of the “National Civilized City” and conducted heterogeneous discussions around the size of urban population, urban financial strength, and tourism city. It provided reference for accurately grasping the policy effect differences of the “National Civilized City” selection among regions. Moreover, this research attempted to deeply explore the internal mechanisms of the impact of civilized city construction on urban inclusive development from macro, meso, and micro levels, providing theoretical and empirical explanations for promoting urban inclusive development.

## 3. Policy background and theoretical analysis

### 3.1 Policy background

**Fig 1:**
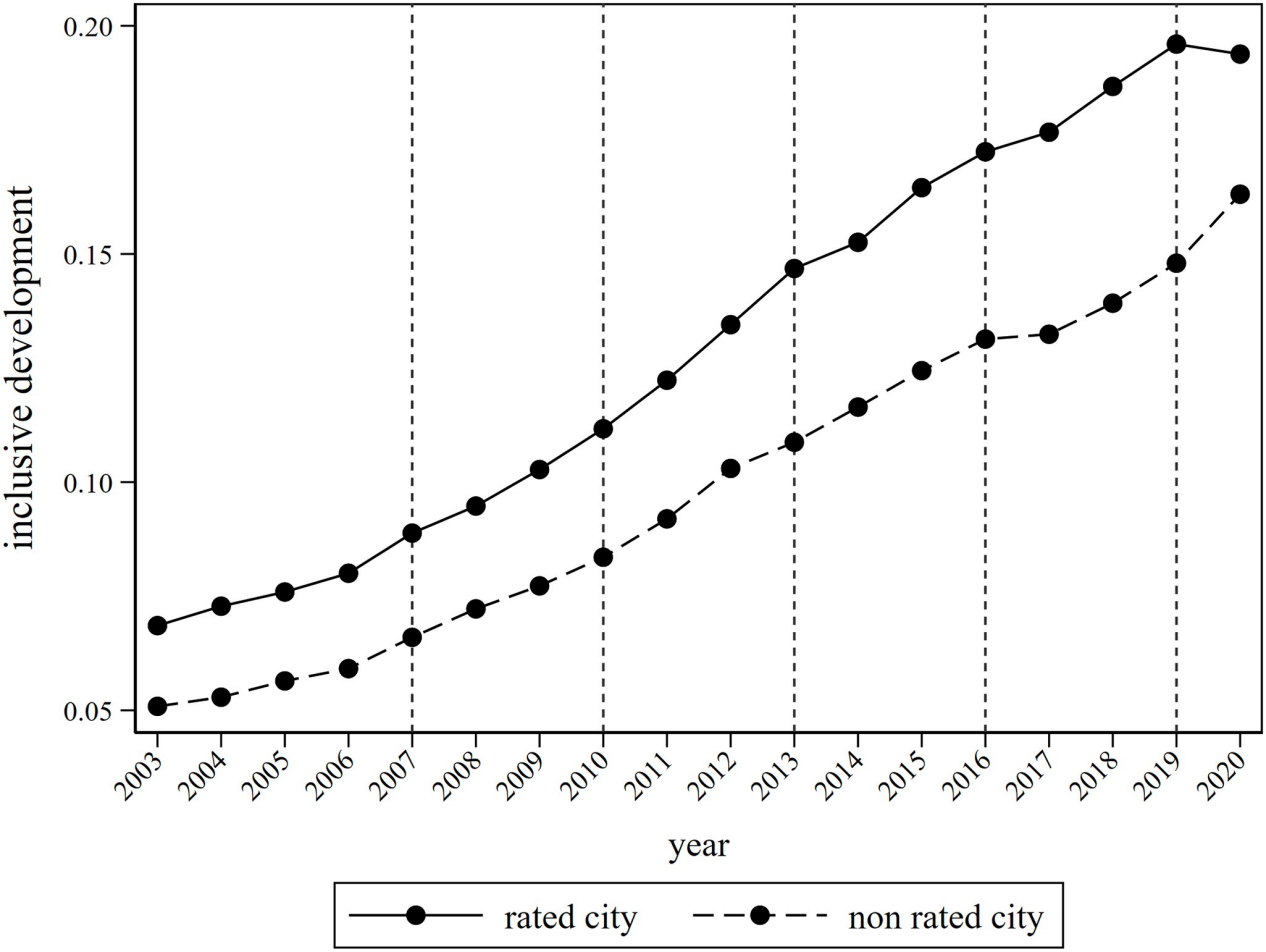
Trends in urban inclusive development between rated and non rated cities from 2003 to 2020.

Since the reform and opening up, China’s urbanization had been rapidly advancing, and the material living conditions have gradually improved. However, the construction of spiritual civilization had been seriously ignored. Thus, in order to meet the growing demand for spiritual civilization among the people, the Central Guiding Committee for the Construction of Spiritual Civilization (hereinafter referred to as the Central Civilization Commission) was established in 1997. In 2003, the Central Commission for Civilization officially launched the “National Civilized City” selection and recognition work, with the main purpose of improving individual overall quality, promoting urban civilization construction, and promoting urban development at a higher level, striving to build a harmonious and livable civilized city. The “National Civilized City” implemented a deadline system and was selected every three years. Since its launch in 2003, a total of six evaluations had been conducted. The characteristics of batch selection have created conditions for the establishment of the multi time point double difference model in the following text. Firstly, the prerequisite for participating in the selection of civilized cities was to obtain the title of “National Advanced City in Creating Civilized Cities” (changed to “Nominated City for Creating National Civilized Cities” after 2008). Then, the application was generally submitted by the governments of various regions, and the superior government and the Central Commission for Civilization provide review opinions. Finally, the list of elected cities was announced. In the process of selecting civilized cities, a highly detailed “National Civilized City Evaluation System” had gradually been formed, which includes two main parts: basic indicators reflecting the creation of civilized cities and characteristic indicators reflecting the characteristics of spiritual civilization creation work and the overall image of the city. Compared with other city honor evaluations, this system is more comprehensive and reliable. At the same time, in order to ensure the sustainability of civilized city construction, the Central Commission for Civilization began implementing a reevaluation mechanism in 2011, which means that the evaluated cities are regularly reviewed every year, and problematic areas are given warnings or even revoked honorary titles. To investigate the impact of civilized city construction on urban inclusive development, this article calculates the level of urban inclusive development based on the following text, and plots the inclusive development trends of rated and non-rated cities, as shown in Fig 1. Among them, the vertical dashed line represents the year of policy impact. It can be seen that compared to non-rated cities, the inclusive development level and growth rate of rated cities are significantly higher. However, this does not provide direct evidence for the construction of civilized cities to drive inclusive urban development, and rigorous theoretical analysis and empirical testing are still needed.

### 3.2 Theoretical analysis

#### 3.2.1 The impact of civilized city construction on urban inclusive development

As a new goal and major development strategy pursued by various cities, urban inclusive development mainly includes three levels: equal development opportunities, comprehensive development content, and shared development results [33]. First, in terms of equality of development opportunities, the construction of a national civilized city will help to create a harmonious and stable social atmosphere, ensure that all social subjects enjoy equal status in the ownership of means of production, and ensure the fairness of development opportunities by expanding public service investment, thus contributing to the realization of inclusive urban development. Second, in terms of the comprehensiveness of development content, the Regional Population, Resources, Environment, and Development Coordination System (PRED System) is a classic interpretation of the comprehensiveness of development content. The construction of civilized cities not only enhances the level of urban civilization, but also further improves the urban population structure, improves resource utilization efficiency through optimizing resource allocation, and has a positive impact on pollution control and ecological environment optimization [17]. It truly promotes the coordinated development of the urban PRED system from the dimensions of population, land, economy, society, and ecology, reflecting the comprehensiveness of development content. Third, in terms of the sharing of development achievements, on the one hand, the construction of civilized cities helps to enhance the personal literacy of residents, cultivate the spirit of tolerance, to a certain extent solve disputes caused by uneven distribution of interests, and narrow the distribution gap; On the other hand, it will also provide more employment opportunities for low-income groups, improve their income levels, and narrow the income gap. At the same time, the construction of civilized cities emphasizes the creation of a fair and just legal environment, which helps to promote fairness in public policy formulation and the human nature of public governance, and further realizes the sharing of development achievements from the perspective of government management. In addition, in order to create a comfortable living environment, the construction of civilized cities also places greater emphasis on the accessibility of public service supply and the benefit of public resource utilization, greatly promoting the sharing of development achievements. Based on this, this article proposes hypothesis 1.

**H1**: **Civilized city construction can promote inclusive urban development**.

#### 3.2.2 The heterogeneity of civilized city construction’s impact on urban inclusive development

Given the inherent differences in economic development levels and fiscal revenue and expenditure among regions, there is much heterogeneity in the impact of civilized city construction on urban inclusive development. First, consider the heterogeneity of urban population size. The heterogeneity of population size that affects the inclusive development of cities through the construction of civilized cities is mainly reflected in the agglomeration and diffusion effects. On the one hand, the larger the urban population size, the more conducive it is to enhancing the agglomeration effect, leveraging the economies of scale and industrial advantages brought about by the construction of civilized cities, attracting the influx of high-quality rural labor, significantly narrowing the income gap between urban and rural residents, and achieving inclusive urban development by providing equal development opportunities and ensuring the sharing of development achievements [34]; On the other hand, the larger the urban population size, the more conducive it is to unleash the diffusion effect. Relying on the free flow of population to accelerate the transfer of factors between urban and rural areas, it promoted the coordinated development of surrounding areas, and thereby ensuring equal opportunities for regional development, achieving inclusive urban development. Meanwhile, cities with larger populations have significant demographic dividends, which can effectively reduce the urban-rural income gap and promote inclusive urban development [35]. Second, consider the heterogeneity of urban financial strength. Fiscal expenditure is an important means of redistribution to narrow income disparities and achieve inclusive development. The stronger the financial strength of the government, the more it can fully play its redistributive role, thereby ensuring the equalization of basic public services, narrowing income disparities, and promoting the sharing of development achievements. At the same time, local finance is an important guarantee for the government to achieve its functions and carry out public activities, and the creation of a “National Civilized City” naturally cannot do without strong support from local finance. The significant investment in human, material, and financial resources in the construction of civilized cities comes from the financial revenue of local governments. Cities with strong financial strength can provide strong financial support for “City Creation” activities, ensure the smooth development of public policy formulation, public service supply, and public resource allocation, accelerate the pace of civilized city construction, ensure the comprehensiveness of development content, and effectively promote inclusive urban development. However, cities with insufficient financial strength will face financial constraints in carrying out “City Creation” work, and various tasks will be difficult. Even if the current work is ensured through overdraft finance, it will still have a negative impact on the inclusive development of cities in the future. Third, consider whether it is an excellent tourism city with heterogeneity. Developing excellent tourism cities is to promote the development of the city’s tourism industry, improve the modern tourism function of the city, and promote economic development and social progress. Compared to non-excellent tourism cities, excellent tourism cities need to further optimize the development environment of the tourism industry and enhance city awareness, and urban brand construction precisely meets the above needs. As the most valuable city brand, “National Civilized City” helps to enhance the reputation and influence of the city, attracting more tourists from other places by releasing signals, and promoting inclusive development of the city through the economic and population mobility effects of tourism development. Based on this, this article proposes hypothesis 2.

**H2**: **The impact of civilized city construction on urban inclusive development varies depending on the size of the city’s population, financial strength, and whether it is an excellent tourist city**.

#### 3.2.3 The internal mechanism of the impact of civilized city construction on urban inclusive development

The impact of civilized city construction on urban inclusive development will be achieved through mechanisms at macro, meso, and micro levels. First, from a macro perspective, the construction of civilized cities promotes inclusive urban development through government public regulations. The construction of civilized cities is a public service that is related to the common interests of all people and benefits the overall society. As the core and main body of public service management, the government affects the progress and effectiveness of civilized city construction. Therefore, the construction of civilized cities will encourage local governments to continue increasing general public budget expenditures to enhance the level of urban social security and employment support, improve urban health conditions, maintain urban public safety, and enhance the government’s level of public regulation. The above-mentioned government public regulation measures, through the readjustment effect of income distribution, have comprehensively improved the living and medical conditions of low-income groups in terms of public services, public resources, public management, and other aspects. They have played an irreplaceable role in ensuring equal development opportunities, promoting comprehensive development content, and achieving the sharing of development achievements. Thus, from the creation of a "national civilized city" to achieving truly inclusive urban development, it is inseparable from strong government public regulatory measures to support it. Second, from a meso perspective, the construction of civilized cities achieves inclusive urban development by promoting industrial structure upgrading. The selection of “National Civilized Cities” has put forward stricter requirements for the quality of urban ecological environment, constantly forcing industrial departments to improve resource utilization efficiency through technological upgrading, structural optimization, and other means. While deepening the connection between traditional and emerging industries, it is also further expanding industrial scale, promoting industrial agglomeration, and achieving the transformation and upgrading of industrial structure. On the one hand, the upgrading of industrial structure will bring about the expansion of the scale of non-agricultural industries, especially the rapid development of the service industry. By absorbing a large number of rural surplus labor to increase farmers’ wage income, it is beneficial to narrow the urban-rural income gap and promote inclusive urban development [36]; On the other hand, the upgrading of industrial structure has led to the rapid growth of the service industry and the transfer of low-end manufacturing industries. By leveraging the sinking migration effect, it provides inclusive employment opportunities for rural labor force that has not yet been transferred and low-quality labor force that has been excluded from the manufacturing industry. Moreover, the contribution of technological innovation brought about by the upgrading of industrial structure to agricultural progress has entered a higher level, further improving agricultural production efficiency, and promoting the sharing of development achievements, To achieve inclusive urban development. Third, from a micro level perspective, the construction of civilized cities promotes inclusive urban development by strengthening talent aggregation and infrastructure construction. Firstly, the construction of civilized cities can leverage the signal effect of urban brands, and achieve inclusive urban development by enhancing the level of talent aggregation. As the city brand that reflects the overall level of civilization in China with the highest value, “National Civilized City” can reflect the openness and inclusivity of the city, promote the signal effect of the rated city, convey the atmosphere of urban growth to high-quality talents with migration intentions, win the favor of more high-quality talents, and thereby enhance the level of talent aggregation in the city. The new culture and ideas brought by the floating population, especially high-level talents, further enhance the inclusiveness of cities and provide effective support for promoting inclusive development of cities. In addition, talent aggregation can also, under the effect of circular accumulation, promote the attraction of more talents to civilized cities, achieve self-strengthening of talent aggregation level, and further enhance the level of urban inclusive development. Second, the construction of a civilized city can improve the material civilization of the city, and provide material guarantee for promoting the inclusive development of the city by strengthening infrastructure construction. In the construction of civilized cities, higher requirements and standards have been put forward for public infrastructure such as cultural and sports facilities, road facilities, and accessibility facilities, greatly strengthening the construction of urban infrastructure. The sound infrastructure will drive the coordinated development of surrounding areas through radiation effects, enhance the equality of development opportunities, further achieve the sharing of development achievements, and promote the inclusive development of cities. Based on the above analysis, this article proposes hypothesis 3.

**H3**: **The mechanism of the role of civilized city construction in the inclusive development of cities is reflected at three levels: macro, meso, and micro**.
**H3a**: **At the macro level, the construction of civilized cities promotes inclusive urban development by strengthening government public regulations**.
**H3b**: **At the meso level, the construction of civilized cities achieves inclusive urban development by promoting industrial structure upgrading**.
**H3c**: **At the micro level, the construction of civilized cities promotes inclusive urban development by strengthening talent aggregation and infrastructure construction**.

## 4. Research design

### 4.1 Model building

#### 4.1.1 Benchmark regression model

According to http://www.wenming.cn/, the list of national civilized cities (districts) published in this article summarizes 146 cities at or above the prefecture level from the six evaluated lists. Due to the incomplete evaluation system for the “National Civilized City” selection in 2005, the first batch of 9 cities that were evaluated excluded from both the processing and control groups. At the same time, it was rated in the first half of the year, and this article sets it as having the title of “National Civilized City” that year. The evaluation time is in the second half of the year, and it is set to have the title of “National Civilized City” in the following year. In addition, considering that applying for a national civilized city requires obtaining the title of “National Advanced City in Creating Civilized Cities”, which is usually obtained 2–3 years before the official evaluation, this article refers to the practice of Wu et al. (2015) and believes that the cities participating in the selection have already been impacted by policies two years before being awarded the title of “National Civilized City”. Thus, this article ultimately sets the impact time for the selection of the second to sixth batches of “National Civilized Cities” as 2007, 2010, 2013, 2016, and 2019, respectively.

When evaluating the effectiveness of policies, the double difference method is a widely used econometric method in recent years. Its core idea is to view institutional changes or the implementation of new policies as an exogenous quasi natural experiment. The selection of “National Civilized Cities” may lead to differences in the inclusive development level of the same evaluated city before and after policy shocks, and may also lead to differences between evaluated and non-valuated cities at the same time point. Using the double difference method (DID) to study the impact of civilized city construction on urban inclusive development can eliminate the temporal and spatial effects of civilized city construction, solve endogeneity problems that may arise from the mutual influence between civilized city construction and urban inclusive development, and more accurately evaluate the impact of civilized city construction on urban inclusive development. Thus, this article regards the selection of “National Civilized Cities” as a quasi-natural experiment and constructs a multi time point double difference model (DID) based on the above dual differences to evaluate the impact of civilized city construction on urban inclusive development. The specific model setting is shown in Eq (1):

$$inclu_{it}=\beta_{0}+\beta_{1}did_{it}+\gamma X_{it}+\mu_{i}+\lambda_{t}+\varepsilon_{it}$$ (1)

Where, *i* and *t* represent the *i*-th city and the *t*-th year, respectively; *inclu*_*it*_ indicates the level of inclusive development in cities; *X*_*it*_ represents a control variable; *μ*_*i*_ and *λ*_*t*_ represent individual and time fixed effects, respectively; *ε*_*it*_ is a random perturbation term. *did*_*it*_ is the i-th city was awarded the title of “National Civilized City” in year *t* or not. The coefficient *β*_1_ is the double difference estimator, which represents the degree of impact of the construction of a civilized city on the inclusive development of the city.

#### 4.1.2 Mediating effect model

Based on the above theoretical mechanism analysis, in order to test the internal mechanism of the impact of civilized city construction on urban inclusive development, this paper constructs a mesomeric effect model as shown in Formula (2) and (3):

$$M_{it}=\beta +\varphi did_{it}+\gamma X_{it}+\mu_{i}+\lambda_{t}+\varepsilon_{it}$$ (2)

$$inclu_{it}=\beta_{0}+\beta_{1}did_{it}+\beta_{2}M_{it}+\gamma X_{it}+\mu_{i}+\lambda_{t}+\varepsilon_{it}$$ (3)

Where, *M*_*it*_ is the mediating variables replaced with macro, meso, and micro level mediating variables in sequence. Coefficient *φ* and *β*_2_ are significant, indicating that the mesomeric effect is established. Meanwhile, the coefficient *β*_1_ is also significant and the symbol matches *φ*×*β*_2_ consistent, it further indicates that the model has some mesomeric effect, and the proportion of mesomeric effect in the total effect is *φ*×*β*_2_/(*φ*×*β*_2_+*β*_1_).

### 4.2 Model building

#### 4.2.1 Dependent variable: Urban inclusive development (*inclu*)

The research referred to the research of Zhang and Wang [37], and constructs a comprehensive evaluation index system for urban inclusive development from three levels: equal development opportunities, comprehensive development content, and shared development results, as shown in Table 1. Meanwhile, this article uses the entropy method to calculate the weight of each indicator to measure the level of urban inclusive development.

**Table 1:** Comprehensive evaluation index system for urban inclusive development.

Criteria layer		Indicator layer	Unit	Attribute	Weight
Equal development opportunities		Per capita paved road area	m^2^/ person	+	0.0560
		Number of hospital beds per thousand people	Zhang/1000 people	+	0.0836
		Per capita public financial expenditure on education	yuan	+	0.1827
		Per capita public finance technology expenditure	yuan	+	0.1058
Comprehensive development content	Population development	The proportion of non agricultural employees	%	+	0.0028
		Urban registered unemployment rate	%	-	0.0002
	Environmental protection	Energy consumption per 10000 yuan of GDP	Standard coal/10000 yuan	-	0.0002
		Harmless treatment rate of household waste	%	+	0.0586
	Economic development	Per capita Gross Regional Product	yuan/ person	+	0.1523
		The ratio of added value to GDP in the tertiary industry	%	+	0.0257
Sharing of development achievements		The ratio of per capita disposable income of urban residents to per capita net income of rural residents	-	-	0.0114
		Ratio of per capita consumption expenditure between urban and rural residents	-	-	0.0012
		Per capita green space area	m^2^	+	0.3196

#### 4.2.2 Core explanatory variable: Dummy variable for the selection of “National Civilized Cities” (*did*)

This variable referred to whether a city has been awarded the title of “National Civilized City”. Specifically, *did*_*it*_ = *treat*_*i*_ × *post*_*t*_, if a city is awarded the title of “National Civilized City”, *treat*_*i*_ = 1; otherwise 0 will be taken; If the time is after being awarded the title of “National Civilized City”, *post*_*t*_ = 1, the remaining time will be taken as 0. It is worth noting that for cities that have not passed the review, this article believes that they will no longer be affected by policy *post*_*t*_ = 0, and will be given a value of 1 until the title is restored.

#### 4.2.3 Control variable

Referring to existing literature [38], this article selects the following five urban level control variables that may affect the construction of civilized cities and the inclusive development of cities for analysis: (1) Population agglomeration level (*lnden*). Population agglomeration is an important support for economic vitality, which helps to promote urban inclusive development by giving play to scale effect and agglomeration effect. This paper uses population density to measure, and takes natural logarithm to deal with it; (2) Economic openness level (*fore*). The improvement of economic openness level helps to promote the development of international trade, and affects the inclusive development of cities by optimizing industrial layout and improving international competitiveness. This article uses the proportion of total import and export trade to GDP to express; (3) Financial development level (*fin*). A sound financial system will provide sufficient financial support for the construction of civilized cities and alleviate financing constraints during the construction process. This article measures the proportion of year-end deposit balances of financial institutions to GDP; (4) Resident savings level (*lnsave*). The improvement of savings level will not only accumulate funds to support the construction of a civilized city, but also affect the investment and consumption level of residents, thus affecting the inclusive development of the city. This paper uses the balance of savings deposits of urban and rural residents per capita at the end of the year, and takes natural logarithm to deal with it; (5) Internet Development Level (*lnnet*). As an important symbol of the information age, the Internet can realize the rapid spread of new knowledge and technology, thus changing the inclusive development process of cities. The research measured the number of Internet users per 10000 people, and takes natural logarithm for processing.

#### 4.2.4 Mediating variable

At the macro level, this article uses the proportion of social security and employment expenditure to fiscal expenditure (*security*), and the proportion of health expenditure to fiscal expenditure (*health*) as the characterization variables of government public regulation.

At the meso level, the investigation drawed on the research of Yuan and Zhu [39] used the relative changes in the proportion of each industry share to measure the upgrading of industrial structure (*stru*). The specific calculation method is shown in Eq (4):

$$stru_{it}=\sum_{m=1}^{3}y_{imt}\times m,m=1,2,3$$ (4)

Where, *y*_*imt*_ represents the proportion of the *m*-th industry in the *i*-th region to the regional GDP during period *t*.

At the micro level, considering the availability of data, this article measures the level of urban talent agglomeration (*talent*) by the sum of the number of professionals in scientific research, technical services, and geological exploration industries, as well as the number of professionals in information transmission, computer services, and software industries, as a proportion of the total number of urban unit employees; This article uses highway mileage divided by regional area to represent highway density (*load*) to measure the level of infrastructure construction.

### 4.3 Data source and processing

Considering the availability of data, the investigation identified the research object as prefecture level and above cities from 2003 to 2020. Due to the participation of various urban areas in Beijing, Shanghai, Tianjin, and Chongqing in the “National Civilized City” selection, rather than the entire city, the above four municipalities were excluded. After further excluding the first batch of evaluated cities, this article ultimately determined sample data from 272 prefecture level and above cities for research. The list of winners was compiled by the Central Civilization Network. The original data for other variables were sourced from the 2004–2021 China Urban Statistical Yearbook, EPS database, and statistical yearbooks of cities at and above the local level. Some missing data were supplemented using interpolation and linear trend methods.

## 5. Research design

### 5.1 Descriptive statistics

Based on the panel data of 272 cities at prefecture level and above in China from 2003 to 2020, this paper analyzes the impact of civilized city construction on urban inclusive development, and the descriptive statistical results are shown in Table 2. It can be observed that there are significant differences in the level of urban inclusive development, with a minimum of 0.0215 and a maximum of 0.6443. The average value of the experimental group (tree) is 0.4890, indicating that 48.90% of cities are in the experimental group, and 51.10% are in the control group. The average DID is 0.1634, indicating that 16.34% of the sample cities have been awarded the title of “National Civilized City”. At the same time, the overall standard deviation of all variables is small, indicating that the sample statistics are relatively close to the overall parameter values, and using sample statistics to infer overall parameters has high credibility.

**Table 2:** Descriptive statistics.

Variable	Meaning of variable	Mean	Mean standard deviation	Minimum	Maximum
*inclu*	urban inclusive development	0.1141	0.0574	0.0215	0.6443
*treat*	policy implementation dummy variable	0.4890	0.4999	0.0000	1.0000
*did*	dummy variable for the selection of “National Civilized Cities”	0.1634	0.3698	0.0000	1.0000
*lnden*	population agglomeration level	5.6870	0.9104	1.5479	8.3571
*fore*	economic openness level	0.0180	0.0210	0.0000	0.3759
*fin*	financial development level	1.2945	0.6075	0.2452	20.1002
*lnsave*	resident savings level	15.6820	1.0834	10.8214	19.0074
*lnnet*	internet Development Level	2.2048	1.1081	-5.1071	5.0031
*security*	the proportion of social security and employment expenditure to fiscal expenditure	0.1270	0.0494	0.0030	0.4191
*health*	the proportion of health expenditure to fiscal expenditure	0.0811	0.0326	0.0013	0.3892
*stru*	the upgrading of industrial structure	0.2775	0.2119	0.0002	3.4301
*talent*	the level of urban talent agglomeration	0.0269	0.0156	0.0000	0.1462
*load*	the level of infrastructure construction	0.9192	0.5084	0.0284	2.6279

### 5.2 Benchmark regression results

Table 3 lists the benchmark regression results of the impact of civilized city construction on urban inclusive development. Among them, column (1) reports the regression results after only considering year and city fixed effects; The second column reports the regression results after considering the control variables; The (3) and (4) columns respectively report the results of the “National Civilized City” selection and the previous, selected, and future returns.

**Table 3:** Impact of civilized city construction on urban inclusive development.

Variable	(1)	(2)	(3)	(4)
*did*	0.0139***	0.0126***	0.0099***	0.0101**
	(0.0010)	(0.0009)	(0.0011)	(0.0041)
*lnden*		-0.0060*	-0.0082**	-0.0662***
		(0.0035)	(0.0041)	(0.0074)
*fore*		-0.1785***	-0.0680***	-0.1871**
		(0.0181)	(0.0149)	(0.0767)
*fin*		-0.0038***	-0.0031***	0.0024
		(0.0007)	(0.0005)	(0.0044)
*lnsave*		-0.0074***	0.0019*	-0.0629***
		(0.0015)	(0.0011)	(0.0094)
*lnroad*		-0.0024***	-0.0007	-0.0142***
		(0.0007)	(0.0005)	(0.0044)
Constant term	0.1118***	0.2752***	0.1232***	1.7000***
	(0.0003)	(0.0313)	(0.0283)	(0.1708)
Fixed year effect	Control	Control	Control	Control
Urban fixed effects	Control	Control	Control	Control
Sample size	4 896	4 896	4 186	710
Adjusted R^2^	0.9108	0.9140	0.9240	0.9249

Note:

***, **, * respectively represent significance levels of 1%, 5%, and 10%, with robust standard errors within ().

The benchmark regression results show that regardless of whether the control variable is considered or not, the estimated coefficients representing the policy variable are all positive and pass the significance test at the 1% level, preliminarily confirming the hypothesis that civilized city construction can promote urban inclusive development; Both before and after the evaluation, the estimated coefficients of *DID* are significantly positive at the 1% level, and the estimated coefficients after evaluation are slightly higher than before, indicating that the construction of civilized cities has indeed promoted urban inclusive development and enhanced the credibility of benchmark regression results. With the implementation of the work to create a “National Civilized City”, the overall civilization of Chinese cities and the personal literacy of residents have significantly improved, the equalization of basic public services has been effectively guaranteed, the distribution gap and income gap have further narrowed, and the development status of cities has been improved from all aspects such as equal development opportunities, comprehensive development content, and shared development results, promoting inclusive urban development. Based on this, hypothesis 1 is proven.

### 5.3 Parallel trend test

The multi time point double difference model needs to satisfy the parallel trend assumption, that is, before policy implementation, the inclusive development of pilot cities and non-pilot cities should have the same trend of change. For this reason, this paper refers to the event study proposed by Jacobson et al. to conduct parallel trend test [40], and the construction model based on Eq (1) is shown in Eq (5):

$$inclu_{it}=\beta_{0}+\sum_{t=-5,t\neq -1}^{7}\delta_{t}D_{it}+\gamma X_{it}+\mu_{i}+\lambda_{t}+\varepsilon_{it}$$ (5)

Where, *D*_*it*_ is a series of dummy variables. If city *i* has been determined as a national civilized city in year *t*, the value is 1, and vice versa, it is 0. The symbolic meanings of the other variables are consistent with Eq (1). The research will focus on the coefficient *δ*_*t*_, which reflects the difference in inclusive development between pilot cities and non-pilot cities in the *t*-th year of being awarded the title of “National Civilized City”.

**Fig 2:**
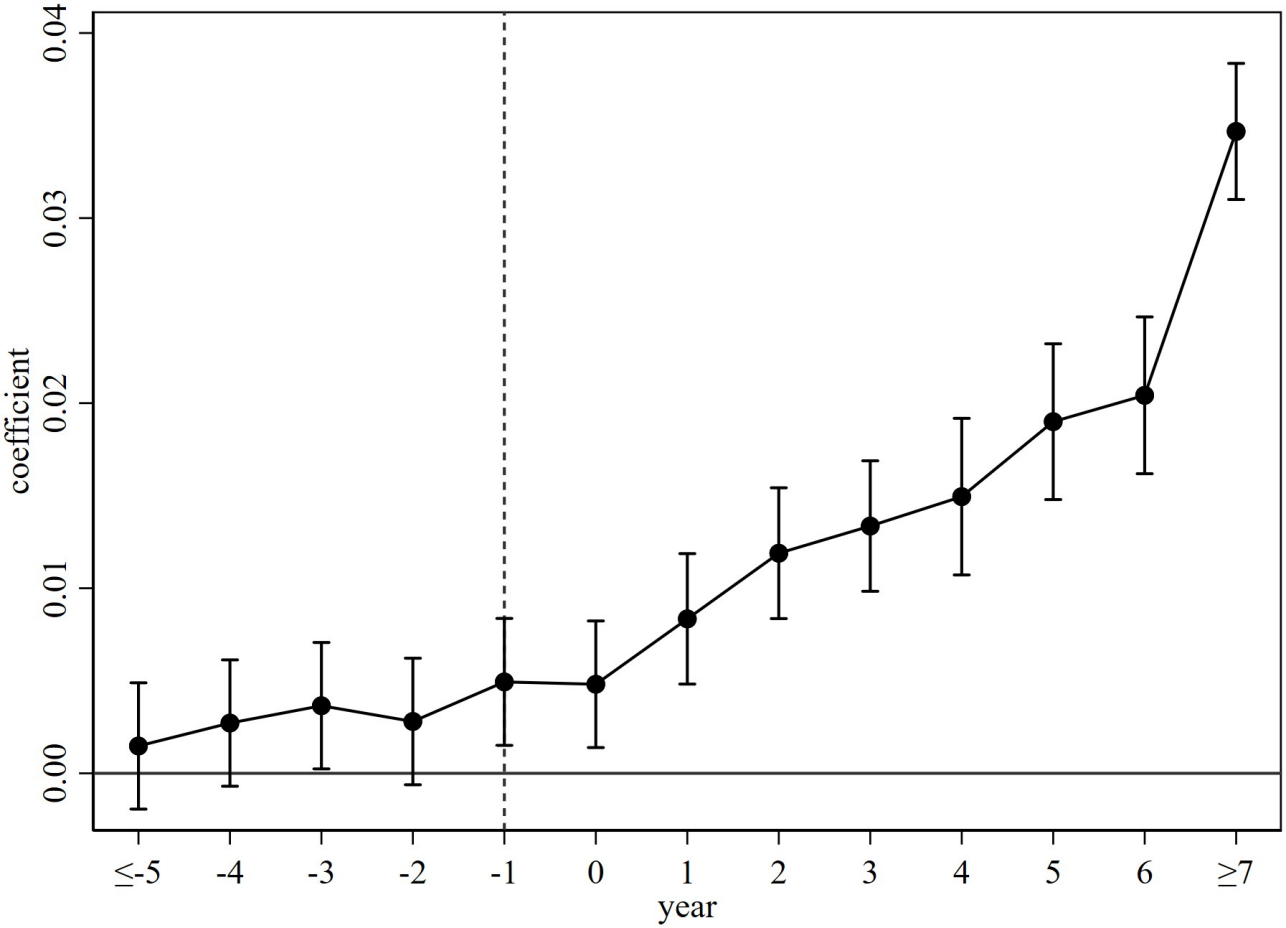
Parallel trend test.

Due to the limited amount of data before the 5th issue and after the 7th issue, this article summarizes the data before the 5th issue to the 5th issue, and summarizes the data after the 7th issue to the 7th issue, with the 1st issue as the base period for analysis. Fig 2 shows the parallel trend test results, showing the estimated values of the coefficients and their 95% confidence intervals. The results show that the *δ*_*t*_ before policy implementation is basically not significant, indicating that there is no significant difference in the inclusive development level between civilized and uncivilized cities in China before policy implementation, and the parallel trend test is passed.

### 5.4 Placebo test

**Fig 3:**
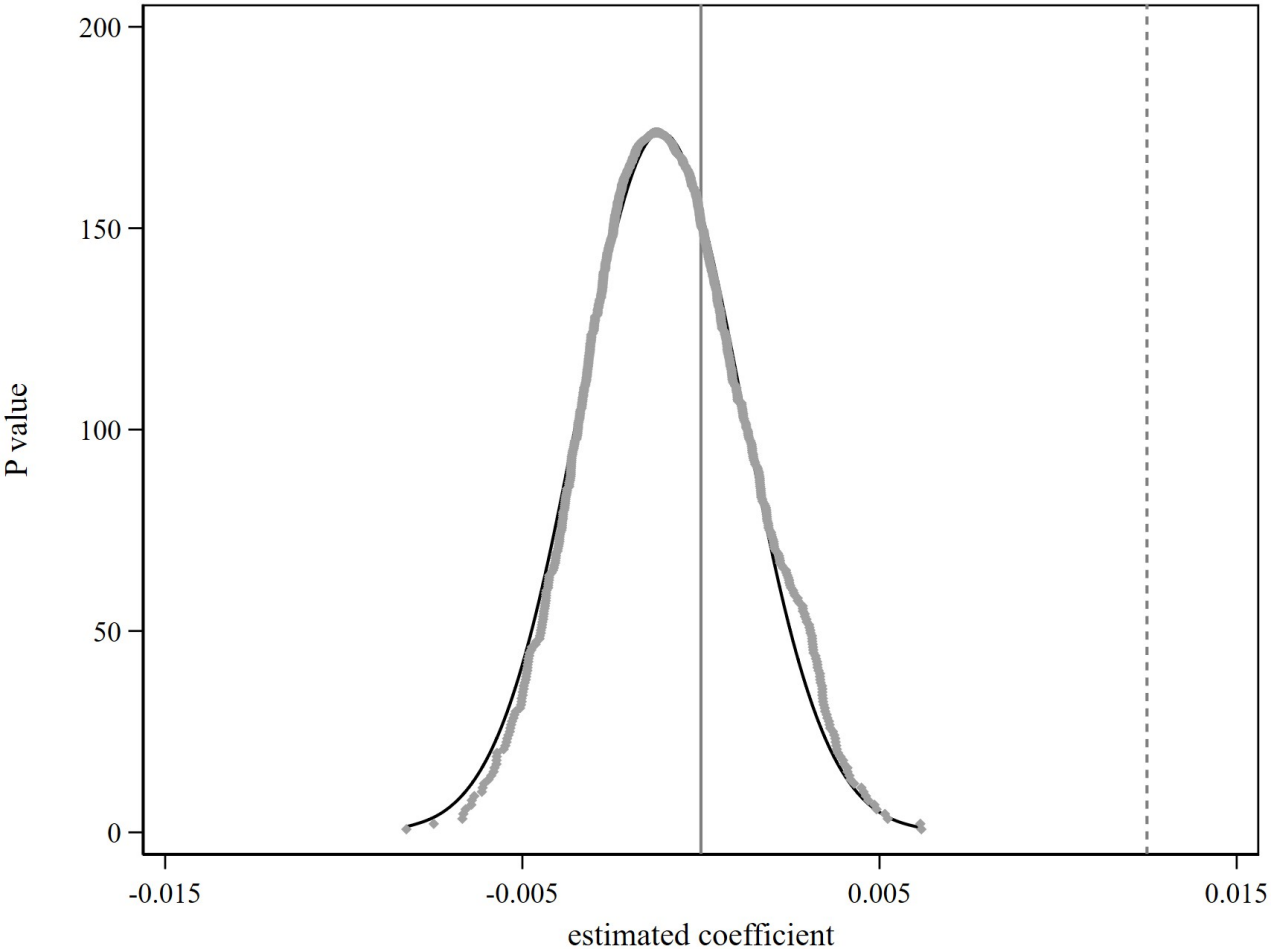
Placebo test.

In order to avoid the impact of other omitted variables on the benchmark regression results, the research conducted a placebo test by replacing the urban samples of the experimental group [41]. Randomly select 133 cities from the sample cities as the false experimental group cities, and the remaining cities as the false control group cities. Use Eq (1) to obtain the estimated coefficients of the impact of civilized city construction on urban inclusive development in sequence. Repeat the above process 1000 times and draw the kernel density curve of the estimated coefficient, as shown in Fig 3. In the figure, the vertical dashed line represents the estimated coefficient value of the benchmark regression results. It can be seen that the estimated value of the regression coefficient is close to 0 and approximately follows the Normal distribution. The estimated value of the coefficient of the benchmark regression is far greater than the estimated value of the false regression coefficient, which is in line with the expectations of the placebo test. This further indicates that other missing variables have not affected the promotion of civilized city construction on the inclusive development of the city, and verifies the authenticity of the benchmark regression results.

### 5.5 Other robustness tests

Although the benchmark regression results appear statistically significant enough to validate the hypothesis that the construction of civilized cities promotes inclusive urban development, there is still a high possibility of estimation bias in the regression results due to the lack of consideration of sample selection bias, endogeneity issues, and various other confounding factors. Thus, the robustness of the benchmark regression results will be further verified through analysis from four dimensions: propensity score matching model, narrowing sample interval, non randomness test, and excluding interference from other policies (Li and Wang, 2016). The results are shown in Table 4.

**Table 4:** Robustness Test I.

Variable	PSM-DID		Reduce sample interval	Non randomness test
	Kernel matching	Radius matching		
	(1)	(2)	(3)	(4)
*did*	0.0123***	0.0122***	0.0095***	0.0109***
	(0.0010)	(0.0010)	(0.0021)	(0.0010)
*lnden*	-0.0311***	-0.0312***	-0.0156	
	(0.0032)	(0.0032)	(0.0100)	
*fore*	-0.2159***	-0.2137***	-0.0709**	
	(0.0188)	(0.0190)	(0.0325)	
*fin*	-0.0108***	-0.0108***	-0.0028	
	(0.0012)	(0.0012)	(0.0019)	
*lnsave*	-0.0078***	-0.0080***	-0.0039	
	(0.0015)	(0.0016)	(0.0062)	
*lnnet*	-0.0052***	-0.0051***	-0.0016	
	(0.0015)	(0.0015)	(0.0013)	
*lnden*×*Trend*				-0.0000***
				(0.0000)
*fore*×*Trend*				-0.0001***
				(0.0000)
*fin*×*Trend*				-0.0000***
				(0.0000)
*lnsave*×*Trend*				-0.0000***
				(0.0000)
*lnnet*×*Trend*				-0.0000
				(0.0000)
*Z*_*1*_×*Trend*				0.0015***
				(0.0002)
*Z*_*2*_×*Trend*				0.0004
				(0.0003)
Constant term	0.4798***	0.4832***	0.2782**	-0.0053
	(0.0337)	(0.0340)	(0.1169)	(0.0426)
Fixed year effect	Control	Control	Control	Control
Urban fixed effects	Control	Control	Control	Control
Sample size	4 884	4 888	3 330	4 896
Adjusted *R*^*2*^	0.9271	0.9250	0.9284	0.9156

After propensity score matching, the positive promoting effect of civilized city construction on urban inclusive development is still significant at the 1% level, preliminarily verifying the robustness of the benchmark regression results. Considering the interaction between various variables and time trend terms, the regression coefficient of civilized city construction is still significantly positive at the 1% level, and the estimated coefficient is not significantly different from the benchmark regression results, indicating that there is no non randomness problem and verifying the robustness of the regression results.

In order to avoid the superposition effect of the implementation of other policies on urban inclusive development during the study period, and thus the error of the benchmark estimation results, the smart city pilot policy, innovative city pilot policy and low-carbon city pilot policy closely related to this study are selected to eliminate possible interference.

Among them, *wisdom*, *inno*, and *low* respectively indicate whether a certain city belongs to the smart city pilot, innovative city pilot, and low-carbon city pilot. If yes, take 1, and vice versa, take 0. Based on Eq (1), this article sequentially incorporates the dummy variables of the three policies mentioned above into the regression, while considering the interference of the three policies. The regression results are shown in Table 5. The results indicate that the pilot policy of "National Civilized City" can promote inclusive urban development independently of other policies. From the estimated coefficients of DIDs in columns (1)-(4) of Table 5, it can be seen that after incorporating and simultaneously considering the three urban pilot policies, the impact of the “National Civilized City” pilot policy on urban inclusive development is still significantly positive at the 1% level. From the estimated coefficients of other policies, it can be seen that the three pilot policies also have a promoting effect on urban inclusive development at a significance level of 1%. This indicates that the benchmark regression results are robust, and the promoting effect of civilized city construction on urban inclusive development is not affected by other policies.

**Table 5:** Robustness Test II.

Variable	Smart city pilot policy	Pilot policies for innovative cities	Pilot policies for low-carbon cities	Three pilot policies for cities
	(1)	(2)	(3)	(4)
*did*	0.0121***	0.0109***	0.0119***	0.0102***
	(0.0010)	(0.0010)	(0.0010)	(0.0010)
*wisdom*	0.0038***			0.0033***
	(0.0010)			(0.0010)
*inno*		0.0105***		0.0098***
		(0.0013)		(0.0013)
*low*			0.0061***	0.0049***
			(0.0013)	(0.0013)
*lnden*	-0.0064*	-0.0077**	-0.0075**	-0.0091***
	(0.0035)	(0.0034)	(0.0035)	(0.0034)
*fore*	-0.1830***	-0.1605***	-0.1771***	-0.1644***
	(0.0181)	(0.0181)	(0.0180)	(0.0181)
*fin*	-0.0038***	-0.0037***	-0.0038***	-0.0037***
	(0.0007)	(0.0007)	(0.0007)	(0.0007)
*lnsave*	-0.0073***	-0.0075***	-0.0071***	-0.0072***
	(0.0015)	(0.0015)	(0.0015)	(0.0015)
*lnnet*	-0.0024***	-0.0017**	-0.0023***	-0.0016**
	(0.0007)	(0.0007)	(0.0007)	(0.0007)
Constant	0.2753***	0.2842***	0.2782***	0.2862***
	(0.0312)	(0.0311)	(0.0312)	(0.0310)
Fixed year effect	Control	Control	Control	Control
Urban fixed effects	Control	Control	Control	Control
Observation	4 896	4 896	4 896	4 896
Adjusted *R*^*2*^	0.9142	0.9151	0.9144	0.9156

During the study period, the COVID-19 outbreak severely affected inclusive urban development. To this end, the years 2020 and 2019 will be excluded in order to control the impact of COVID-19 on the baseline regression results. The regression results are shown in Table 6. The results show that the impact of civilized city construction on inclusive urban development is still significantly positive at the level of 1%, indicating that the impact of the novel coronavirus epidemic is excluded, and the robustness of the baseline regression results is verified.

**Table 6:** Robustness Test III.

Variable	Excluding 2020	Excluding 2019 and 2020
	(1)	(2)
*did*	0.0131***	0.0148***
	(0.0022)	(0.0028)
*lnden*	0.0540**	0.0425*
	(0.0270)	(0.0239)
*fore*	-0.1776***	-0.1697***
	(0.0599)	(0.0565)
*fin*	-0.0027	-0.0060
	(0.0020)	(0.0038)
*lnsave*	-0.0088	-0.0069
	(0.0054)	(0.0052)
*lnnet*	-0.0029**	-0.0024*
	(0.0015)	(0.0013)
Constant	-0.0479	-0.0140
	(0.1383)	(0.1258)
Fixed year effect	Control	Control
Urban fixed effects	Control	Control
Observation	4 624	4 352
Adjusted *R*^*2*^	0.9122	0.9104

### 5.6 Heterogeneity analysis

The above study analyzed the overall impact of civilized city construction on urban inclusive development. However, this analysis of the sample population may overlook potential regional differences. Hypothesis 2 discusses that the impact of civilized city construction on urban inclusive development may differ significantly depending on the size of the city’s population, financial strength, and whether it is an excellent tourist city.

#### 5.6.1 Heterogeneity of urban population size

From the perspective of population size, there are significant differences in infrastructure construction, human capital level, and other aspects among cities of different sizes. Therefore, it is worth exploring whether the construction of civilized cities will exhibit differentiated policy effects due to the different population sizes of cities. Referring to the Notice on Adjusting the Standards for Urban Scale Classification released by the State Council, cities at or above the prefecture level are divided into large cities and small and medium-sized cities based on whether their urban permanent population reaches 1 million. The virtual variable of large cities is recorded as 1, and small and medium-sized cities are recorded as 0.

On this basis, the interaction term between the urban population size dummy variable (people) and the selection dummy variable (did) is added to the benchmark regression model to explore whether the policy effect of civilized city construction will differ due to different urban population sizes. The results are shown in Table 7. The results show that did and did × The estimated coefficients of people are significantly positive at the 1% level, indicating that the construction of civilized cities has a promoting effect on the inclusive development of both large and small and medium-sized cities, but it has a stronger promoting effect on large cities. Column (2)-(4) in Table 7 show that the construction of civilized cities can promote equal development opportunities, comprehensive development content and sharing of development achievements of big cities and small and medium-sized cities, but the promotion effect of big cities is stronger.

**Table 7:** Heterogeneity of urban population size.

Variable	*inclu*	equal development opportunities	comprehensive development content	sharing of development achievements
	(1)	(2)	(3)	(4)
*did*	0.0115***	0.0055***	0.0039***	0.0001***
	(0.0010)	(0.0003)	(0.0003)	(0.0000)
*did*×*people*	0.0138***	0.0013***	0.0018*	0.0001**
	(0.0031)	(0.0001)	(0.0011)	(0.0000)
*lnden*	-0.0054	-0.0051***	0.0168***	0.0001***
	(0.0035)	(0.0012)	(0.0012)	(0.0001)
*fore*	-0.1805***	-0.0727***	-0.0697***	0.0012***
	(0.0180)	(0.0061)	(0.0062)	(0.0003)
*fin*	-0.0039***	-0.0015***	-0.0017***	-0.0001***
	(0.0007)	(0.0002)	(0.0002)	(0.0000)
*lnsave*	-0.0071***	-0.0017***	-0.0043***	0.0002***
	(0.0015)	(0.0005)	(0.0005)	(0.0000)
*lnnet*	-0.0024***	-0.0011***	-0.0013***	0.0000***
	(0.0007)	(0.0002)	(0.0002)	(0.0000)
Constant	0.2677***	0.0843***	-0.0018	0.0000
	(0.0313)	(0.0106)	(0.0107)	(0.0005)
Fixed year effect	Control	Control	Control	Control
Urban fixed effects	Control	Control	Control	Control
Observation	4 896	4 896	4 896	4 896
Adjusted *R*^*2*^	0.9143	0.8594	0.8711	0.8886

#### 5.6.2 Heterogeneity of urban financial strength

From the perspective of financial strength, the construction of civilized cities cannot be separated from the support of government finance, so the impact of civilized city construction on urban inclusive development may vary depending on the government’s financial strength. The financial strength of the government is measured by the local general Public budgeting revenue consisting of taxes, central allocation and land use right sales. For this reason, the average of general Public budgeting revenue of each city in the study period is calculated, and the city dummy variable greater than the median is recorded as 1, and the other cities are recorded as 0. Furthermore, the interaction term between the fiscal strength dummy variable (*revenue*) and the selection dummy variable (*did*) was added to the benchmark regression model, and the results are shown in Table 8.

**Table 8:** Heterogeneity of urban financial strength.

Variable	*inclu*	equal development opportunities	comprehensive development content	sharing of development achievements
	(1)	(2)	(3)	(4)
*did*	0.0102***	0.0024***	0.0020***	-0.0001***
	(0.0019)	(0.0006)	(0.0006)	(0.0000)
*did*×*revenue*	0.0033***	0.0038***	0.0026***	-0.0000
	(0.0010)	(0.0007)	(0.0007)	(0.0000)
*lnden*	-0.0064*	-0.0055***	0.0164***	0.0001***
	(0.0035)	(0.0012)	(0.0012)	(0.0001)
*fore*	-0.1763***	-0.0702***	-0.0677***	0.0012***
	(0.0181)	(0.0061)	(0.0062)	(0.0003)
*fin*	-0.0038***	-0.0015***	-0.0018***	-0.0001***
	(0.0007)	(0.0002)	(0.0002)	(0.0000)
*lnsave*	-0.0074***	-0.0017***	-0.0044***	0.0002***
	(0.0015)	(0.0005)	(0.0005)	(0.0000)
*lnnet*	-0.0023***	-0.0010***	-0.0012***	0.0000***
	(0.0007)	(0.0002)	(0.0002)	(0.0000)
Constant	0.2773***	0.0864***	0.0010	-0.0000
	(0.0313)	(0.0106)	(0.0107)	(0.0005)
Fixed year effect	Control	Control	Control	Control
Urban fixed effects	Control	Control	Control	Control
Observation	4 896	4 896	4 896	4 896
Adjusted *R*^*2*^	0.9141	0.8602	0.8714	0.8885

The results show that *did* and *did*×*revenue* estimated coefficients of revenue are significantly positive at the 1% level, indicating that regardless of the city’s financial strength, the construction of a civilized city will contribute to the inclusive development of the city, but it has a stronger promoting effect on cities with strong financial strength. Column (2)-(4) in Table 8 show that the construction of civilized cities can promote equal development opportunities and comprehensive development content of cities with strong financial strength.

#### 5.6.3 Heterogeneity of excellent tourism cities

Compared to non-excellent tourism cities, excellent tourism cities have a stronger dependence on urban brands. Therefore, as the most valuable urban brand, “National Civilized City” may have varying degrees of impact on the inclusive development of cities between excellent and non-excellent tourism cities. Thus, whether it has been awarded the title of "China’s Excellent Tourism City" will be used as the grouping basis. The virtual variable of excellent tourism cities will be recorded as 1, and non-excellent tourism cities will be recorded as 0. On the basis of the benchmark regression model, the interaction term between the virtual variable of excellent tourism cities (travel) and the selected virtual variable (did) will be added. The results are shown in column (3) of Table 9.

**Table 9:** Heterogeneity of excellent tourism cities.

Variable	*inclu*	equal development opportunities	comprehensive development content	sharing of development achievements
	(1)	(2)	(3)	(4)
*did*	0.0087***	0.0031***	0.0022**	0.0001**
	(0.0027)	(0.0009)	(0.0009)	(0.0000)
*did*×*travel*	0.0052**	0.0025***	0.0021**	0.0002***
	(0.0028)	(0.0010)	(0.0010)	(0.0000)
*lnden*	-0.0060*	-0.0052***	0.0166***	0.0002***
	(0.0035)	(0.0012)	(0.0012)	(0.0001)
*fore*	-0.1784***	-0.0720***	-0.0688***	0.0012***
	(0.0181)	(0.0061)	(0.0062)	(0.0003)
*fin*	-0.0038***	-0.0016***	-0.0018***	-0.0001***
	(0.0007)	(0.0002)	(0.0002)	(0.0000)
*lnsave*	-0.0074***	-0.0016***	-0.0044***	0.0002***
	(0.0015)	(0.0005)	(0.0005)	(0.0000)
*lnnet*	-0.0024***	-0.0010***	-0.0013***	0.0000***
	(0.0007)	(0.0002)	(0.0002)	(0.0000)
Constant	0.2752***	0.0844***	-0.0002	-0.0001
	(0.0313)	(0.0106)	(0.0107)	(0.0005)
Fixed year effect	Control	Control	Control	Control
Urban fixed effects	Control	Control	Control	Control
Observation	4 896	4 896	4 896	4 896
Adjusted *R*^*2*^	0.9140	0.8592	0.8712	0.8891

The results show that *did* and *did*×*travel* estimated coefficients of travel are significantly positive at the 1% level, indicating that the construction of civilized cities has a significant promoting effect on the inclusive development of both excellent and non-excellent tourism cities, but it has a stronger promoting effect on excellent tourism cities. Column (2)-(4) in Table 9 show that the construction of civilized cities can promote equal development opportunities, comprehensive development content and sharing of development achievements of excellent tourism cities. In summary, hypothesis 2 is proven.

## 6. Mechanism verification

In order to deeply understand the internal mechanism of civilized city construction to promote urban inclusive development, based on the Mesomeric effect model and theoretical mechanism analysis, the impact of civilized city construction on urban inclusive development is analyzed from the macro level of government public regulation, the meso level of industrial structure upgrading, and the micro level of talent gathering and infrastructure construction.

### 6.1 Macro level: Government public regulation

At the macro level, the construction of civilized cities affects the inclusive development of cities through government public regulations. This article replaces the intermediate variable (*M*_*it*_) with the proportion of social security and employment expenditure to fiscal expenditure (*security*) and the proportion of health expenditure to fiscal expenditure (*health*). The regression results are shown in Table 10. Among them, the estimated coefficients of *did* in columns (1) and (2) are significantly positive at the 1% level, indicating that the construction of civilized cities has significantly increased social security and employment investment, as well as health investment, indicating that the construction of civilized cities has significantly improved the government’s level of public regulation. The improvement of the government’s level of public regulation will improve the living and medical conditions of low-income groups [9], thereby contributing to achieving inclusive urban development. Based on this, it is assumed that H3a is proven.

**Table 10:** Mechanism testing.

Variable	Macro level		Meso level	Micro level	
	(1)	(2)	(3)	(4)	(5)
	*security*	*health*	*stru*	*talent*	*load*
*did*	0.0114***	0.0031***	0.0093***	0.0015***	0.0436***
	(0.0015)	(0.0009)	(0.0012)	(0.0004)	(0.0091)
*lnden*	-0.0212***	0.0048	-0.0949**	0.0089***	0.0463
	(0.0056)	(0.0032)	(0.0453)	(0.0015)	(0.0334)
*fore*	-0.0978***	0.1374***	-1.8374***	0.0162**	0.1447
	(0.0293)	(0.0167)	(0.2362)	(0.0076)	(0.1742)
*fin*	0.0073***	0.0001	0.1610***	0.0004	-0.0163**
	(0.0012)	(0.0007)	(0.0095)	(0.0003)	(0.0070)
*lnsave*	-0.0113***	-0.0029**	-0.0836***	-0.0047***	0.0659***
	(0.0024)	(0.0014)	(0.0197)	(0.0006)	(0.0145)
*lnnet*	0.0000	0.0036***	-0.0254***	-0.0013***	0.0451***
	(0.0011)	(0.0006)	(0.0088)	(0.0003)	(0.0065)
Constant	0.4172***	0.0892***	2.6557***	0.0513***	-0.4649
	(0.0508)	(0.0290)	(0.4093)	(0.0132)	(0.3019)
Fixed year effect	Control	Control	Control	Control	Control
Urban fixed effects	Control	Control	Control	Control	Control
Observation	4 896	4 896	4 896	4 896	4 896
Adjusted *R*^*2*^	0.6933	0.7717	0.9296	0.7943	0.8978

### 6.2 Meso level: Government public regulation

In order to investigate the mechanism of the role of industrial structure upgrading in the impact of civilized city construction on urban inclusive development, this article replaces the intermediary variable (*M*_*it*_) with industrial structure upgrading (*Stru*), and the regression results are shown in columns (3) of Table 10.

The results show that the estimated coefficient of *did* in column (3) is significantly positive at the 1% level, indicating that the construction of civilized cities has effectively promoted the upgrading of industrial structure. Upgrading the industrial structure will provide more inclusive employment opportunities (Yan and Wang, 2020) [42], improve agricultural production efficiency, and ultimately achieve urban inclusive development. Thus, assuming H3b is proven.

### 6.3 Micro level: Talent aggregation and infrastructure construction

In order to further explore the mechanism of talent agglomeration in the impact of civilized city construction on urban inclusive development, this paper replaces the intermediary variable (*M*_*it*_) with talent agglomeration level (*talent*) to test the Mesomeric effect. The results are shown in Table 10, Column (4). Column (4) reports the estimated results of the impact of civilized city construction on talent aggregation. The results show that the estimated coefficient of *did* is significantly positive at the 1% level, indicating that the construction of civilized cities has played a role in talent aggregation. The improvement of talent aggregation level will bring new ideas and new culture, and through the cumulative effect of circulation, continuously enhance the level of urban inclusive development. Based on this, the intermediary mechanism for talent gathering has been proven (Yao et al., 2023) [15].

In order to further improve the internal mechanism at the micro level, and focus on discussing the role of infrastructure construction in the impact of civilized city construction on urban inclusive development, this paper will replace the intermediary variable (*M*_*it*_) with the road density (*load*) to test the Mesomeric effect. The results are shown in Table 10, Column (5). The results show that the estimated coefficient of did in column (5) is significantly positive at the 1% level, indicating that the construction of civilized cities has effectively improved the level of urban infrastructure construction. A well-developed infrastructure can drive the coordinated development of surrounding areas, enhance the equality of development opportunities, and ultimately achieve urban inclusive development Thus, the intermediary mechanism for infrastructure construction has been proven.

## 7. Conclusion and policy recommendation

### 7.1 Conclusion

Accurately grasping the policy effect of the “National Civilized City” selection on urban inclusive development has important theoretical and practical significance for improving the level of urban civilization and promoting urban inclusive development. Based on the quasi-Natural experiment selected by “National Civilized Cities”, the research takes 272 cities at prefecture level and above in China from 2003 to 2020 as research samples, and uses the multi time point double difference model to systematically assess the impact of civilized city construction on urban inclusive development.

It can be concluded that the construction of civilized cities significantly promotes inclusive urban development. The analysis before and after the evaluation further confirms the effectiveness of the above effects, and parallel trend tests, placebo tests, and other robustness tests all confirm the reliability of the research conclusions. Heterogeneity analysis found that the impact of civilized city construction on urban inclusive development varies depending on the size of the city’s population, its financial strength, and whether it is an excellent tourist city. That is, in areas with large population size, strong financial strength, and belonging to excellent tourist cities, civilized city construction has a stronger promoting effect on urban inclusive development. Mechanism testing found that the construction of civilized cities mainly promotes inclusive urban development through macro level government public regulations, meso level industrial structure upgrading, and micro level talent aggregation and infrastructure construction.

### 7.2 Policy recommendation

Based on the above research conclusions and the current situation of civilized city construction and urban inclusive development, this article proposes the following suggestions:

Firstly, continue to promote the construction of civilized cities and improve the inclusive development environment of cities. Although “creating a city” is a long process that requires a large amount of manpower, material resources, and financial resources, this study found that the construction of civilized cities effectively promotes inclusive development of cities. Therefore, it is necessary to encourage various cities to participate in the selection, improve the overall level of urban civilization, and play a promoting role in inclusive development of cities, creating a harmonious environment for sharing the achievements of the development of a moderately prosperous society. In addition, cities that have been rated should develop strict regulatory measures to prevent major social problems and stabilize the achievements of “City creation”.

Secondly, we must adhere to adapting measures to local conditions and refine the arrangements for the construction of civilized cities based on actual conditions. For cities with small populations and insufficient financial strength, it is necessary to adhere to the phased promotion of civilized city construction, prevent overnight success, and avoid adverse effects on the inclusive development of cities in the future; For outstanding tourism cities that have not been awarded the title of “National Civilized City”, it is necessary to actively carry out the “city creation” work, in order to stimulate the recovery of the tourism industry and promote inclusive development of cities through the use of city brands.

Finally, we should attach importance to the role of government public regulation, industrial structure upgrading, talent gathering, and infrastructure construction, and unblock the impact path of civilized city construction on urban inclusive development. We should actively play the role of the government, continuously increase investment in social security, employment, and health, improve the living environment of low-income groups, and increase labor productivity; Deeply optimize the industrial structure, accelerate the transfer to high value-added service industries, and promote the improvement of urban inclusivity level; Fully leverage the talent gathering effect of civilized city construction, optimize the work and living environment of talents through multiple approaches, attract more talents to flow in and settle down, and further improve infrastructure construction to enhance the material civilization of the city, providing sufficient material guarantee for the inclusive development of the city.

### 7.3 Discussion

This study’s findings on the impact of civilized city construction on urban inclusive development offer a novel perspective in comparison to previous research. While existing literature has acknowledged the importance of urban planning and policy interventions in fostering inclusivity, our empirical analysis using the multi-time point DID model for National Civilized Cities in China over an extended period (2003–2020) provides quantitative evidence that is both robust and granular. The results confirm a significant promotion effect. This research implies that the National Civilized City initiative could serve as a blueprint for other cities aiming to strike a balance between economic progress and social inclusivity.

Despite the robustness tests confirming the reliability of the study, several limitations warrant acknowledgment. First, while the quasi-natural experiment design reduces endogeneity concerns, unobserved factors or omitted variables may still affect the estimated treatment effects. Second, the generalizability of these findings might be limited due to the specific context of Chinese cities. Future research can expand upon these findings by incorporating additional control variables, considering longer time frames, and examining different regional or international contexts. Moreover, studying the potential long-term sustainability and unintended consequences of such policies will also contribute significantly to the body of knowledge on urban development strategies.

## Supporting information

S1 Data (XLSX)
